# Alt a 1 Promotes Allergic Asthma *In Vivo* Through TLR4-Alveolar Macrophages

**DOI:** 10.3389/fimmu.2022.877383

**Published:** 2022-06-30

**Authors:** Guadalupe Hernandez-Ramirez, Diego Pazos-Castro, Zulema Gonzalez-Klein, Jose Luis Resuela-Gonzalez, Sergio Fernandez-Bravo, Lucia Palacio-Garcia, Vanesa Esteban, Maria Garrido-Arandia, Jaime Tome-Amat, Araceli Diaz-Perales

**Affiliations:** ^1^Centre for Plant Biotechnology and Genomics Universidad Politécnica de Madrid, Instituto Nacional de Investigación y Tecnología Agraria y Alimentaria, Consejo Superior de Investigaciones Cientificas (UPM –INIA/CSIC), Universidad Politécnica de Madrid, Madrid, Spain; ^2^Department of Biotechnology-Plant Biology, Escuela Tecnica Superior de Ingeniería Agronómica, Alimentaria y de Biosistemas (ETSIAAB), Universidad Politécnica de Madrid, Madrid, Spain; ^3^Department of Allergy and Immunology, IIS-Fundación Jiménez Díaz, Universidad Autónoma de Madrid (UAM), Madrid, Spain

**Keywords:** allergic asthma, Alt a 1, TLR4, alveolar macrophage, mouse model

## Abstract

The mold *Alternaria alternata* is one of the main sources of asthma exacerbation, being its major allergen, Alt a 1, indispensable for its development. The main objective of this work was to answer two main questions: 1) can Alt a 1 by itself (without any other context) induce an asthmatic profile *in vivo*?; and 2) Which molecular mechanisms take place during this phenomenon? To answer both questions, we have developed a mouse model of allergic asthma using only Alt a 1 for mice sensitization. We also made use of *in-vitro* cellular models and computational studies to support some aspects of our hypothesis. Our results showed that Alt a 1 can induce an asthmatic phenotype, promoting tissue remodeling and infiltration of CD45+ cells, especially eosinophils and macrophages (Siglec F+ and F4/80+). Also, we have found that Alt a 1 sensitization is mediated by the TLR4-macrophage axis.

## 1 Introduction

Asthma is characterized by reversible airway obstruction as the result of predominantly type-2 (T2) driven airway inflammation and pulmonary remodeling. The development of asthma usually starts from repeated environmental allergen exposure (1). Fungal spores constitute the largest proportion of airborne particles, although the majority of mold sensitization research performed points to a few fungal genera to be relevant in airway diseases (2). *Alternaria alternata* is one of the most common aeroallergens sources (3). It has been long described as an outdoor and indoor mold, and a clear risk factor for asthmatic people when spores reach their highest levels in the air during late summer and/or early autumn period (4, 5). Thus, mouse models have been widely used to characterize the ability of *Alternaria* to induce T2 immune responses (3, 6).

Alt a 1 is the only major allergen related to *Alternaria*-induced asthma, being a marker of primary sensitization and chronic asthma (7). Although the biological function of Alt a 1 remains unknown, it seems to show a role in plant pathogenesis (8, 9). Alt a 1 is a small protein (~15 kDa) mainly detected in spores (10) from where it is released in large quantities as a tetramer at pH around 5.0 – 6.5, carrying a flavonol molecule as a ligand (11). Its tetrameric form allows its recognition by SLC22A17, a receptor in bronchial epithelial cells, inducing the production of alarmins such as IL-33 and IL-25 (12). In contrast, Garrido-Arandia et al. also described that this interaction does not affect the permeability of the epithelium (12), and therefore, it cannot be responsible for the entry of Alt a 1 through the epithelium. Thus, there must be another mechanism for the allergen to pass through the epithelium and activate the inflammatory response. Lastly, Alt a 1 has been described to be able to induce innate immunity, depending mainly on TLR4 (6).

Little is known about the mechanisms underlying Alt a 1 sensitization, and many questions remain unanswered, such as the relationship between asthma and Alt a 1, or whether some other component of the spores is necessary helping to induce the T2 response. Similarly, we do not know how Alt a 1 can pass through the epithelium and be presented by the antigen-presenting cells (APC) beneath it, inducing the adaptive response.

The goal of this study has been the characterization of the immunological activity of Alt a 1 *in vivo* by developing a mouse model of allergic asthma. Furthermore, in view of the limited information regarding molecular mechanisms underlying the high prevalence of Alt a 1 sensitization, we also intended to focus on the description of the first interaction between Alt a 1 and the airway environment, extending the interest to the role of alveolar macrophages (AM). In this study, we investigated the innate immunostimulatory activities of Alt a 1 in the lung as well as in a human epithelial bronchial model. We found that Alt a 1 shows a potent cytokine- and chemokine-inducing activity, mostly dependent on Toll-like receptor signaling pathways. Moreover, evidence is shown for a significant interaction between TLR4 and Alt a 1, which could be responsible for TLR dimerization and the activation of the inflammatory cascade.

## 2 Materials and Methods

### 2.1 Mouse Model of Allergic Asthma

Female C57BL/6J mice were obtained from Charles River breeding colony (France); housed in the animal care facilities at IIS-Fundación Jiménez Díaz (Madrid, Spain) under standard laboratory conditions. All animal protocols and procedures were approved by the Institutional Animal Care and Use Committee from Community of Madrid (Ref. PROEX 392/15) and were conducted in compliance with current legislation (European Union Directive 2010/63/EU).

Mice (6–8-week-old) were administered intranasally with recombinant Alt a 1 (12) (50 μg), Complex (50 μg; 1:4 for Alt a 1:quercetin ratio) or saline buffer on days 1, 3, 5, 8 and 11. Control groups of mice that received albumin (BSA or OVA; 50 μg/dose) intranasally were included. Mice were slightly anaesthetized with 3% isoflurane and O_2_ at 1L/min (Abbott Laboratories, North Chicago, IL), and once unresponsive but breathing comfortably, 50 μl of sample solution were directly applied on the nostrils. Mice were sacrificed on day 12.

#### 2.1.1 Humoral Response

The detection of Alt a 1-specific immunoglobulin (Ig) in sera was performed by ELISA and the proper HRP-conjugated antibodies when it was required. Sera were diluted 1:5 for IgE and 1:50 for both IgG bindings (overnight; 4°C). Optical density was determined by measuring the absorbance at 450 nm.

#### 2.1.2 Lung Remodeling

Thin sections (10 - 12 μm) from paraffin embedded mouse lungs were cut with a microtome (Leica RM1235, Wetzlar, Germany), and stained with Haematoxylin/Eosin (H&E; Sigma) or periodic acid-Schiff (PAS; Thermo Fisher) following provider’s instructions.

#### 2.1.3 Bronchoalveolar Lavage (BAL) Cell Analysis

Once the trachea was exposed and incised, a needle (1.2 x 40 mm) was inserted into the trachea and BAL was harvested by rinsing the lungs twice with 1 ml of sterile PBS. Total cell counts were determined with a hemocytometer and the cellular profile of BAL was obtained by flow cytometry using specific antibodies as indicated below. Briefly, after fixation with % PBS-formaldehyde (PFA; 5 min, 25°C), cells were stained with the specific fluorochrome-conjugated antibodies for 1h at RT. After washing, samples from Alt a 1-sensitized mice were analyzed using a MA900 Multi-Application Cell Sorter (Sony Biotechnology, USA). Unstained controls and single stained controls were processed to correct compensation.

#### 2.1.4 Measurement of Lung Function

Lung resistance was measured using a Numiotec ventilator system (Numio Technologies, SL, Madrid, Spain) following provider’s instructions. To this end, inspiration/expiration plots were registered for each mouse for 10 min (3 cycles). Control measurements were obtained over a period of 5 min directly after administration of rocuronium. The average plot was registered for every mouse and the plot represented is the average for each group treated. The maximum point of volume/pressure represents the dynamic compliance and the space between the lower line (inspiration) and upper line (expiration), which is directly related to lung expansion.

#### 2.1.5 Characterization of Lung Cell Infiltrates

To obtain single- cell suspensions, lungs (n = 4/group) were cut into small pieces with blades and digested for 30 min at 37°C in PBS containing 0.1% v/v of BSA (Gibco, Thermo Fisher), 1 mg/ml collagenase A (Roche, Switzerland) and 0.1 mg/ml DNase I (Roche). After, cell aggregates were dissociated, and cells were fixed with 1% PFA for 5 min at RT. Cell sorting was performed on a MA900 (Sony) at a rate allowing minimum 90% of efficiency to recover CD45+ cells. Leucocyte population was subsequently characterized using specific antibodies listed in **Supplementary Table 1**.

#### 2.1.6 Gene Expression Profiling

Lungs were homogenized and lysed with GIT extraction buffer (pH 7; 4 M guanidine isothiocyanate, Sigma-Aldrich; 25 mM sodium citrate, Sigma-Aldrich; 0.5% sarcosyl, Sigma-Aldrich; 0.1 M 2-mercaptoethanol, Carl Roth, Germany). RNA was purified by phenol:chloroform extraction and precipitated with ethanol. cDNA was obtained and a pool of 5 mice/group was analyzed by qPCR using Bio-Rad’s PrimePCR Pathways (Asthma & Allergy, M384; Hercules, CA, USA), following the provider’s instructions. To identify the differentially expressed genes between the Alt a 1 group and the control group, the GeneStudy_1.0.030.1023 software was applied.

#### 2.1.7 Lung Lysates for Western Blotting

Snap-frozen lung tissues were homogenized in RIPA buffer containing 20 mM Tris-HCl (pH 8), 150 mM NaCl, 0.1% SDS, 1% Triton X-100 and EDTA-free protease inhibitor cocktail (Roche). After that, samples were centrifuged for 10 min (4°C) at 10.000 g and supernatants were collected and quantified by Bradford method. Lung lysates were separated by SDS-PAGE (15% acrylamide) and the ORMDL-3 and caspase-1 immunodetections were assured for the use of specific antibodies listed in **Supplementary Table 1**, and the subsequent use of HRP-conjugated anti-IgG antibodies. Anti-tubulin was included to show equal loading and transference of lung protein extracts. The quantification of the bands was carried out by optical densitometry and analyzed using the ImageJ digital imaging processing software (ImageJ 1.53a, National Institutes of Health, Bethesda, MD, USA). The expression of each protein was normalized with β-tubulin signal.

### 2.2 Co-Localization of Alt a 1 With Endosome Markers

To characterize the Alt a 1 traffic, non-polarized Calu-3 cells (ATCC; HTB-54; USA) were grown on coverslips until reached a 70% of confluency. Then, they were incubated with Alt a 1 at 37°C, 5%CO2 for different times: 5 min for early endosome assay, and 30 min for recycling endosome assays. After incubation, cells were washed, fixed with 4% PFA for 10 min and permeabilized with PBS-Triton X100. Cells were blocked and then they were incubated with anti-Alt a 1 antibody and the proper anti-endosome marker (all antibodies listed in **Supplementary Table 1**) for 1h at RT. After using Alexa-conjugated secondary antibodies, cells were stained with DAPI and mounted with ProLong Gold over slides. Images were obtained with a Zeiss LSM 880 confocal microscope, using 405, 488 and 633 laser excitations and 63X amplification.

The recycling pathway was inhibited by adding 100 mM endosidin-2 (ES-2; Sigma-Aldrich) 1h prior to adding Alt a 1 to Calu-3 monolayers. Alt a 1 accumulation was evaluated by immunofluorescence following the earlier described protocol.

### 2.3 Pull-Down Assays

Silica nanospheres (80 nm carboxyl; nanoComposix, California, USA) were conjugated with TLR 4 (Peprotech, New Jersey, USA) in 50 mM MES buffer pH 4.5 containing 0.01% Tween-20 overnight at RT. The functionalized nanoparticles were blocked with 50 mM Tris-HCl pH 8 and casein blocking buffer for overnight at 4°C. For pull-down assays, nanoparticles were incubated with Alt a 1 (0.1 μg/μL) or Complex (0.1 μg/μl) for 2h at room temperature. After centrifugation, pellets were extensively washed and resuspended in denaturing Laemmli buffer. Samples were separated into a 15% SDS‐PAGE and the biding of Alt a 1 or Complex was confirmed by western blot using a specific anti-Alt a 1 antibody. Negative controls of non-functionalized nanoparticles incubated with Alt a 1 or functionalized nanoparticles without Alt a 1 were included.

### 2.4 Lung Immunofluorescence

Intact lung halves (neither treated nor paraffined) from healthy C57BL6/J mice were incubated with Alt a 1 (25 μg) or saline (PBS) for 10 min at 37°C in RPMI 1640 medium (Invitrogen, USA) containing 2 mM L-glutamine, 25 mM HEPES, 10% heat-inactivated fetal bovine serum, and 1% Pen- Strep. Then, lungs were fixed with 4% PFA for 16 h and cut into thin slices. After blocking, lung slices were stained with specific antibodies (listed in **Supplementary Table 1**) for 1h at RT, followed by secondary antibody conjugated with Alexa Fluor Dyes. Nuclei were stained with DAPI, and negative controls of anti IgG-Alexa antibodies were included.

### 2.5 THP1-XBlue-CD14™

The transfected cell line THP1-XBlue-CD14™ (*In vivo*gen, France) derived from the human monocytic THP-1 cell line was used according to the manufacturer’s instructions. Briefly, 1.10 (6) cells/ml were stimulated with Alt a 1 or Complex (5 μg/ml) for 16 h, and, the NF-κB pathway activation was monitored by the detection of the secreted embryonic alkaline phosphatase (SEAP) in the supernatants. For the inhibition tests, cells were incubated with anti-TLR4 antibodies (5 μg/ml; Invitrogen) or an inhibitor of myeloid differentiation protein 2 (10 μM; MedChemExpress, USA) for 15 min prior to the stimulation. In addition, the presence of TNFα and IFNγ was detected in the supernatants from Alt a 1-induced THP1 cells using ELISA kits (ImmunoTools GmbH, Germany), and the results were normalized by the non-stimulated values.

To obtain human macrophages-like cells, THP-1 cells were treated with a final concentration of 100 nM Phorbol 12-Myristate 13-Acetate (PMA; Sigma Aldrich, USA) in RPMI medium for 48h. THP1-derived macrophages were cultured in coverslips and, once they reach a stable morphological state, cells were incubated with Alt a 1 for 2 min at 37°C. Cells were immediately fixed with 4%PFA and TLR4 and Alt a 1-Alexa 647 antibodies were used for an immunofluorescence assay. Nuclei were stained with DAPI and a negative control of secondary antibodies was included. Images were obtained with a Zeiss LSM 880 confocal microscope, using 405, 488 and 633nm laser excitations.

### 2.6 Computational Analysis

Structural comparison between Alt a 1 and myeloid differentiation factor 2 (MD2) was compute using three structural alignment methods implemented on the protein data bank website: FATCAT (13), CE (14) and TM-Align (15). Moreover, it also was analyzed using MMLigner (16), a statistical inference of protein structural alignments and the presence of concepts in the Alt a 1 structure was identified using Procodic (17). The PDB entries 3V0R (18) and 3FXI (19) were used for Alt a 1 and MD2 structure respectively. In order to analyze the interaction between TLR4 and Alt a 1, the initial geometry was obtained with blind docking calculations using ZDOCK (20), and ColabFold (21). Among the models obtained, the initial geometry was chosen based on the similarity of the Alt a 1 localization with respect to MD2 in the TLR4-MD2 crystal structure (PDB: 3FXI).

Alt a 1-TLR4 molecular system and Alt a 1- TLR4 tetramer system was explored with all-atom 50 and 100 ns Molecular Dynamics (MD) simulations using the CHARMM3.1 force field and the multicore CUDA version of NAMD 2.13 in the Tesla V100 GPU of the high-performance computing CBGP. The system was prepared with CHARMM-GUI (22, 23).Periodic solvation boxes with 14 Å spacing in all dimensions and TIP3P (24) water model were used and Na+ and Cl- ions added to counter total charges and set 0.150 M salt concentration. Initial geometries were minimized at 5000 conjugate- gradient optimization steps and water was then equilibrated at 298 K and 1 atm for 100 ps at 2 fs time steps. Production runs were performed during 100 ns simulation time at 2 fs timesteps in the NPT ensemble at 1 atm and 298 K with Langevin dynamics for T control and Nose-Hoover Langevin piston method for P control. Trajectories were processed and analyzed with VMD 1.9.3 (25).The dissociation constant of the TLR4-Alt a 1 and TLR4-MD2 complexes were calculated using Prodigy web server (26, 27).

Molecular graphics were prepared and rendered with UCSF Chimera v1.16 (28). Protein Data Bank the was also analyzed using MMLigner (16). Structures, Alt a 1-TLR4 and MD2-TLR4 tetramer systems were explored with all-atom 100 ns Molecular Dynamics (MD) simulations using the CHARMM 3.1 force field and the multicore CUDA version of NAMD 2.13 in the Tesla V100 GPU of the high-performance computing CBGP. Periodic solvation boxes and water model were used, and Na+ and Cl- ions were added to counter total charges and set 0.150 M salt concentration. Initial geometries were minimized at 5000 conjugate-gradient optimization steps and water was then equilibrated at 298 K and 1 atm for 100 ps at 2 fs timesteps. Production runs were performed during 100 ns simulation time at 2 fs timesteps (50 million steps per simulation) in the NPT ensemble at 1 atm and 298 K with Langevin dynamics for T control and Nos-Hoover Langevin piston method for P control (28).

### 2.7 Statistical Analyses

Statistically significant differences were analyzed by Prism9 (GraphPad Software Inc., La Jolla, CA, USA) using Kruskal-Wallis test with Dunn’s correction for multiple comparisons and P-values < 0.05 were considered significant.

## 3 Results

### 3.1 Alt a 1 Can Trigger Allergic Asthma in a Mouse Model

**Figure 1:**
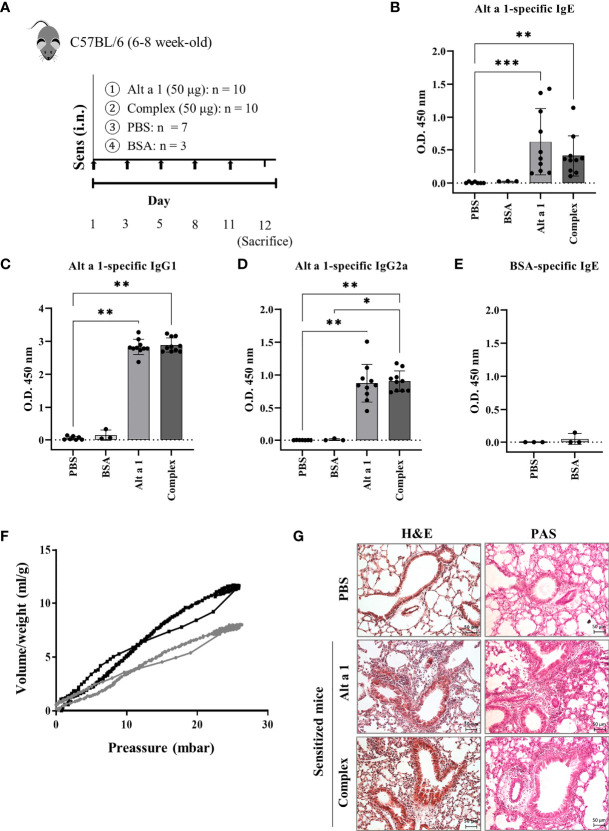
Alt a 1 induces severe allergic asthma. **(A)** Experimental protocol for an allergic airway inflammation model highlighting the timing of Alt a 1, Complex, PBS or BSA intranasal administration into C57BL/6 mice. Measurement of antibody production in serum: **(B)** Alt a 1-specific IgE, **(C)** IgG1 and **(D)** IgG2a. **(E)** Detection of serum BSA-specific IgE in BSA-treated mice. Means-SD are showed (Kruskal-Wallis test; *p<0.05; **p < 0.01; ***p< 0.001). **(F)** Graphic depiction of lung resistance comparing Alt a 1 sensitize mice (grey) and control PBS (black). Each graph represents the average of each group. **(G)** Lung sections were stained with H&E and PAS to study the lung architecture and mucus hypersecretion (bar = 50 mm).

Intranasal exposure of mice to Alt a 1 (**Figure 1A**), in the presence (Complex) or absence (Alt a 1) of its ligand (a quercetin-like molecule), elicited allergic airway inflammation. No differences could be observed as a result of the presence of the ligand. Alt a 1 sensitization was confirmed by measuring specific humoral response (**Figures 1B–D**). This response was only induced by the presence of the allergen and not because of putative environmental pollution, as suggested by the BSA-treated group lacking specific IgE (**Figure 1E**). Moreover, we observed differences in the respiratory capacity between mice with Alt a 1-induced asthma and controls (**Figure 1F**), the first ones also showing severe phenotypic changes in the lung tissue such as sub-epithelial fibrosis, cell infiltration and goblet cell hyperplasia (**Figure 1G**).

**Figure 2:**
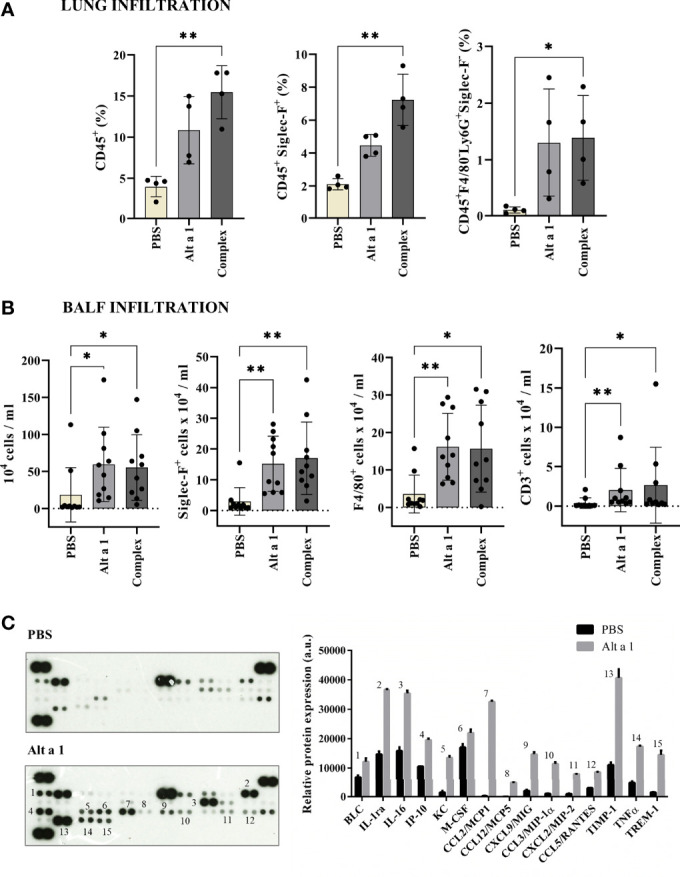
Cell immune infiltrates after Alt a 1 treatment. **(A)** Characterization of CD45+ cell populations from lungs, using Siglec-F, F4/80 and Ly6G markers (n = 4 mice/group). **(B)** Characterization of BALF infiltrates, showing cell counts per lung for total cell number and populations defined by Siglec-F, F4/80 and CD3 markers (n = 10 mice/group; BSA-treated mice were included in control group together non-sensitized mice). Means-SD are showed (Kruskal-Wallis test; *p < 0.05; **p < 0.01). **(C)** Detection of a profile of cytokines in supernatants of BALFs collected from PBS-treated mice (control group) and Alt a 1-treated mice. Data shown are from a twenty minute exposure to X-ray film, using the ImageJ software (version 1.53a) to quantify the signals.

The cell infiltration was mostly due to CD45+ cells, especially macrophages and eosinophils (CD45+SiglecF+) and neutrophils (CD45+ F4/80- Ly6G+ SiglecF-) (**Figure 2A**). Moreover, in the case of the bronchoalveolar lavage fluid (BALF), the high infiltration levels of cells in asthmatic mice was mostly due to eosinophils and AM, (Siglec-F+, F4/80+; **Figure 2B**). The mediator response was an inflammatory remodeling response. When the cytokines present in BALF were quantified (**Figure 2C**), they were mostly inflammatory cytokines (such as TIMP-1 or IL1ra) or chemokines inducing monocyte infiltration (such as CCL2, 12 or CXCL12).

**Figure 3:**
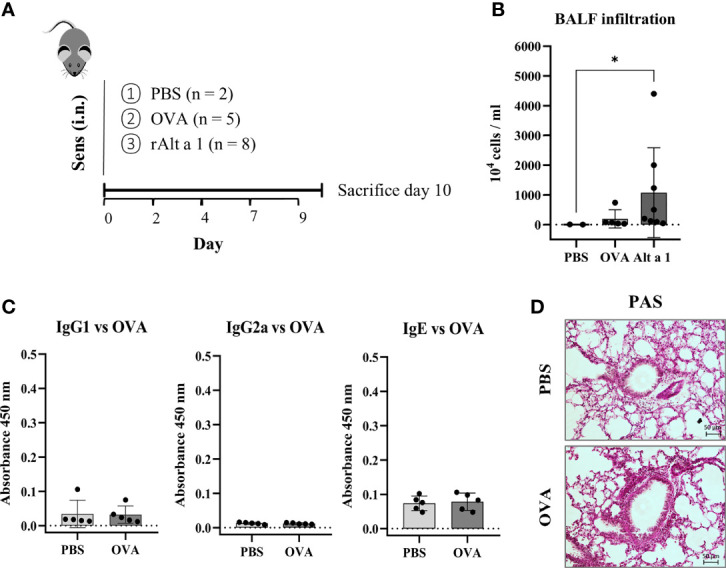
Comparison of mouse model of sensitization using Alt a 1 or Ovoalbumin (OVA). **(A)** Experimental protocol of the mouse model of Alt a 1, Ovoalbumin (OVA) and PBS, through intranasal route into C57BL/6 mice. **(B)** Quantification of total BALF infiltration in the Alt a 1, OVA and PBS mice, expressed as cell concentration in the BALF. **(C)** Measurement of antibody production in serum: OVA-specific IgG1, IgG2a and IgE. Means-SD are showed (Kruskal-Wallis test; *p < 0.05). **(D)** Lung sections stained with PAS comparing lung architecture of OVA and PBS treated mice (bar = 50 μm).

The asthmatic phenotype observed can only be due to the presence of Alt a 1. When another allergen was used as OVA following the same pattern as for the *Alternaria* allergen, no humoral response, no cellular infiltration in the BALF and no alveolar remodeling could be seen (**Figures 3A–D**).

### 3.2 Alt a 1-Induced Asthma Is Characterized by an Increase in the Expression of Asthma Markers

**Figure 4:**
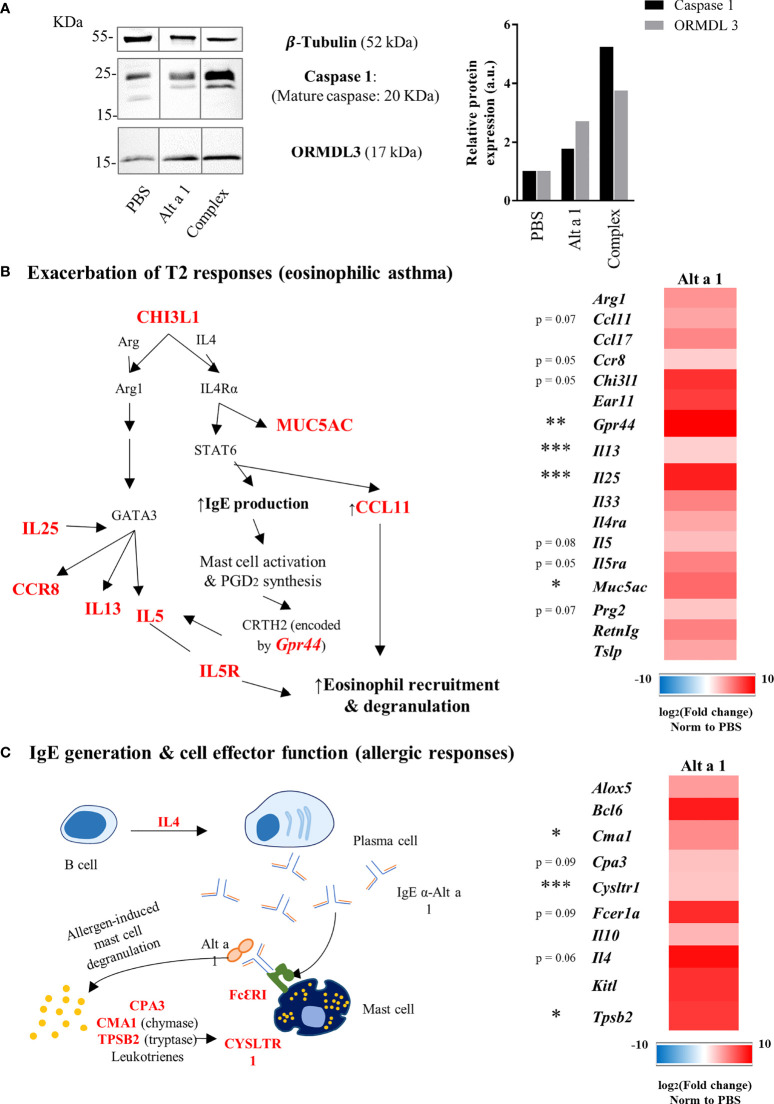
Expression profile of asthma genes induced by Alt a 1. **(A)** Immunodetection of ORMDL- 3 and Caspase 1 in murine lungs. Lung lysates were separate by SDS-PAGE 15% and immunoblotted with the antibodies indicated in the figure. Anti-tubulin was used to show equal loading and and to normalize the signals using ImageJ software version 1.53a for quantification. (b and c) Gene expression profile in lung samples (n = 5 mice/group) was evaluated using a predesigned plate titled “PrimePCR Asthma & Allergy pathways” from Bio-Rad Laboratories. **(B)** Heat map of up-regulated genes related to type 2 responses and a summary diagram showing the interconnections between them. **(C)** Heat map of up-regulated genes related to IgE generation and effector cell functions, also showing a summary diagram to interconnect gene functions. Means-SD are showed (Kruskal-Wallis test; *p < 0.05; **p < 0.01; ***p < 0.001).

The asthmatic phenotype of Alt a 1-sensitized mice was characterized by analyzing the expression of asthma markers such as ORMDL-3 (29) and caspase-1 (30). The presence of both markers was increased in the lungs of asthmatic mice after Alt a 1-sensitization as can be seen in **Figure 4A**. The characterization of the asthmatic profile was completed by transcriptomic analysis study comparing asthmatic versus healthy mice. In this way, 42 genes were found up-regulated (> 4-fold), most of which are related to eosinophilic asthma. The majority of those genes (n=27) corresponds to T2 cytokines and alarmins (i.e., IL25, IL33, IL13); chemokines (i.e., IL5); their receptors (i.e. IL4ra, IL5ra) (**Figure 4B**); or genes related to lung impairment (i.e., Muc5a, Ccll2 or Ccll3) and mast cell functions (i.e., Fcer1a, Tpsb2 or Kitl) (**Figure 4C**).

### 3.3 Alt a 1 Is Recognized and Directed to the Recycling Pathway

**Figure 5:**
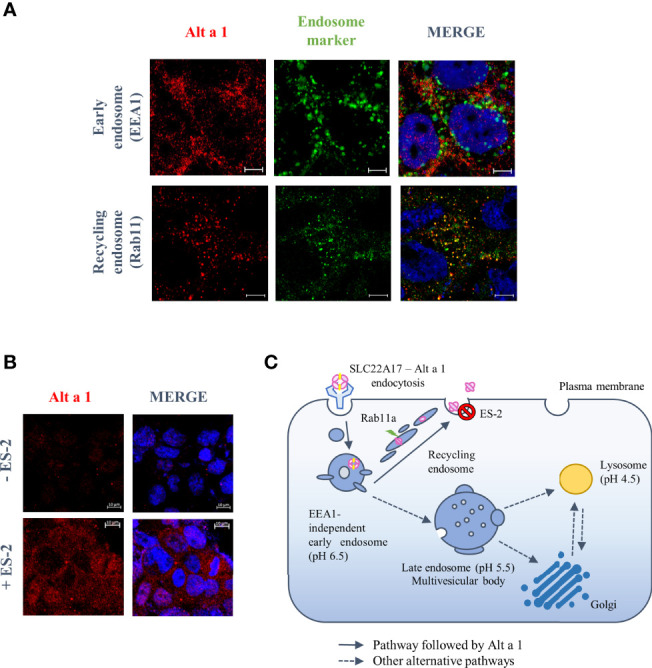
Endosomal trafficking pathway followed by Alt a 1 in Calu-3 cells. **(A)** Immunolocalization of Alt a 1 (red) with EEA1 (green), an early endosome marker, and Rab 11 (green), a recycling endosome marker. Calu-3 cells were incubated with Alt a 1 for 2 min at 37°C to study initial steps of endosome pathway (EEA1-Alt a 1 immunolocalization) and for 20 min at 37°C to study recycling pathway (Rab11-Alt a 1 immunolocalization). Negative controls are included in supplementary figure 1. **(B)** Immunofluorescence to detect Alt a 1 (red) accumulation after inhibition of exocytosis pathway by endosidin-2 (ES-2) application to Calu-3 cells (the signal of cells incubated only with Alt a 1 were used to define the background signal). Bar = 5 mm (except for the section c where the bar = 10 mm). **(C)** Model of endosome pathway followed by Alt a 1 in epithelial cells.

To study how Alt a 1 can pass through the epithelial barrier, an *in vitro* bronchial epithelium model was developed using the human Calu-3 cell line. According to previous data, Alt a 1 can interact with epithelial cells through the SLC22A17 receptor, however the percentage of transported protein is too low to explain the recognition by APCs11. Studying the Alt a 1 internalization into epithelial cells, no co-localization was observed between Alt a 1 and EEA1 (early endosome antigen-1), an early endosome marker, suggesting that protein follows an EEA1-independent pathway. In contrast, Alt a 1 co-localized with Rab 11, a marker of the endosomal-recycling pathway (**Figure 5A**). This result was confirmed through the inhibition with endosidin-2 (ES-2), an exocytosis and endosomal recycling inhibitor (**Figure 5B**). Thus, Alt a 1 could be recycled following the same route as in the case of lipocalin-2, another ligand of SLC22A17 (**Figure 5C**) (31).

### 3.4 Alt a 1 Induces NF-kB Signaling by TLR4 Pathway

**Figure 6:**
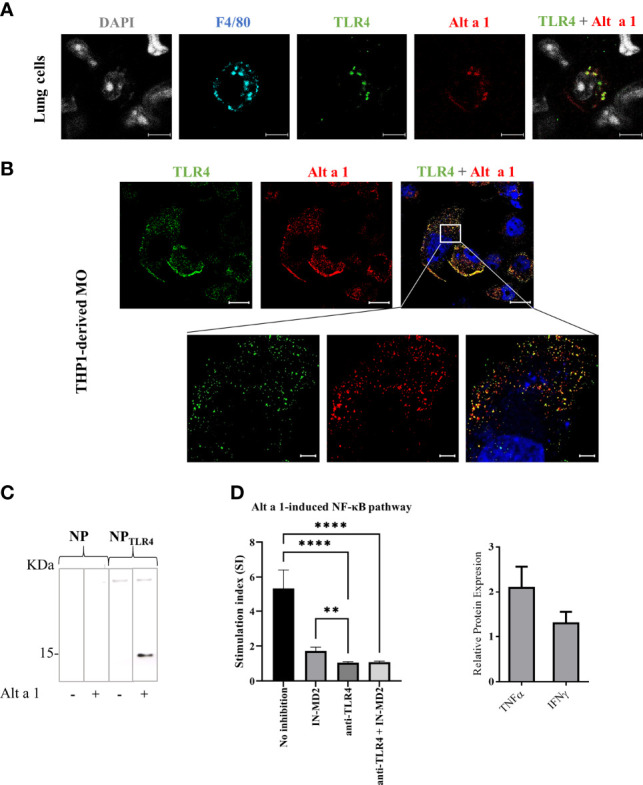
Interaction of Alt a 1 with TLR4. **(A)** Non-fixed lungs immediately after to be removed from healthy mice, were incubated with Alt a 1 for 5 minutes. Then, sections were cut and fixed, and Alt a 1 (red), TLR4 (green) and F4/80 (blue) were immunodetected using specific antibodies labelled with fluorophores. Scale bar = 5 µm. **(B)** THP1-derived macrophages were incubated with Alt a 1 for 2 min at 37°C to localize the protein (red) with TLR4 (green). Scale bar = 20 µm (63x images) and bar= 5 µm (zoom). **(C)** Binding of Alt a 1 to nanoparticles functionalized with TLR4 protein. Nanoparticles were loaded into SDS-PAGE 15% and result was evaluated by western blot using a specific antibody for Alt a 1. **(D)** Activation of NF-kB signaling in cell line THP1-XBlue-CD14+ by Alt a 1 after pre-incubation with TLR4 blockade antibody or MD-2 inhibitor. Stimulation index was calculated as ratio between stimulated cells and non-stimulated cells. Relative protein expression in media culture of THP1 incubated with Alt a 1 compared with PBS control. Data are represented as Mean-SE (Kruskal-Wallis test; **p < 0.01; ****p < 0.0001).

Since the recycling of Alt a 1 might be partially preventing the apical-to-basal transport of the protein through the epithelial barrier, and considering that the protein does not alter the epithelial permeability *in vitro*, the mechanism through which Alt a 1 reaches APCs on the basolateral side remains unclear. One possibility is the recognition of Alt a 1 by AM, which could directly introduce the allergen in the alveoli triggering a pro-inflammatory response, as previously described6. Supporting this hypothesis, co-localization of Alt a 1 with TLR4 in mice lung F4/80+ cells (**Figure 6A**) as well as in THP1-derived macrophages (**Figure 6B**) was observed in immunofluorescence assays. This recognition was also confirmed by pull down-binding assay, using TLR-nanoparticles incubated with Alt a 1 (**Figure 6C**). When Alt a 1 was cultivated with a macrophage cell line, THP1-XBlue-CD14+, the interaction between TLR4 and Alt a 1 was inhibited by adding specific antibodies or specific MD2 inhibitor (32) (**Figure 6D**).

### 3.5 Alt a 1 Shares Structural Homology With MD2 Being Able to Mimic the Role of the Complex MD2-LPS

**Figure 7:**
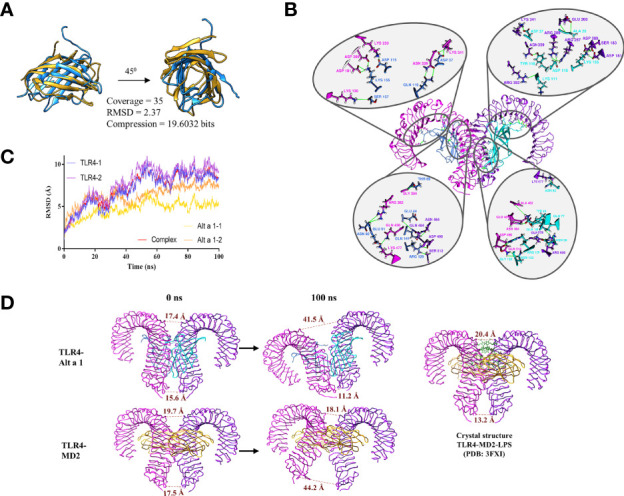
Alt a 1 and its implication in TLR4 dimerization. **(A)** Ribbon representation of the structural comparison between Alt a 1 and MD-2 using MMLigner. **(B)** Alt a 1- TLR4 tetramer was analysed by means of 100ns Molecular Dynamics Simulation. RMSD of the trajectory is shown in the graph, for single proteins and the hole complex. **(C)** Analysis of the three dimensional structure of the tetramer obtained at the end of the simulation. **(D)** Schematic representation of the distance between the alpha carbons of residues Asp 325 (upper part of the structure) and Glu 586 ( bottom part) of the TLR4 structure. Throughout the figure Alt a 1 has been represented in blue, TLR4 in magenta and MD-2 in yellow color.

Based on these results, the structural similarity between Alt a 1 and MD2 was analyzed. Both are small proteins of 15 and 18.5 KDa respectively with a structure characterized by the presence of beta sheets. Although the scores obtained in the similarity analysis do not reveal that they belong to the same protein family, a structural relationship between both can be observed (**Figure 7A**).

To explore whether such structural similarity might be sufficient to complex with TLR4, the interaction between Alt a 1 and TLR4 was explored by means of molecular dynamics simulations using as a reference the interaction between TLR4 and MD2. The results obtained, reveal that the Alt a 1-TLR4 complex remains stable along the simulation (100 ns), as shown by the stabilization of the RMSD values (**Figure 7B**). This complex is maintained by the presence of more than 18 hydrogen bonds (**Figure 7C**). In addition, the distances between the Glu 586 and the Asp 325, in the C terminal region of the TLR4 ectodomain, between each protein of the homodimer were analyzed to know the behavior along the dynamics simulations. It can be seen how in the case of TLR4 with Alt a 1, the distance between the residues is more similar to the crystallographic structure of TLR-MD2-LPS than in the case of TLR4-MD2, in which a high increase could be observed (**Figure 7D**). All these data suggest that Alt a 1 could be bound with high affinity to TLR4, in absence of MD2, promoting the dimerization of the receptor.

## 4 Discussion

*Alternaria alternata* has been reported as one of the most important molds acting as an allergic asthma inducer (10). The present study provides evidence that Alt a 1, regardless of the ligand, promotes airway allergic inflammation in an asthma mouse model. Alt a 1-sensitization induced an airway type 2 inflammation, marked by up-regulation of genes related to eosinophilia and CD45+ infiltration, mucous hypersecretion, and increased of specific antibodies in serum (sIgG1, sIgG2 and sIgE). Even more, Alt a 1-asthmatic mice showed an increase of ORMDL-3 and caspase-1 in the asthmatic mice, both markers related to severe asthma. In the case of ORMDL-3, it has been associated to eosinophil trafficking, their recruitment and degranulation (33, 34), although its expression did not influence in the number of T2 cells. Referring to caspase-1, it can induce the T2 cytokines production inducing the asthma exacerbations. Caspase-1-deficient mice showed no alterations in general lung inflammatory parameters, but a marked reduction in eosinophilia (29). Caspase-1 has been widely studied for promoting the secretion of active forms of IL-1 and IL-18 (35), major contributors to asthma pathogenesis in mice. Focusing on the putative Alt a 1-mechanism to induce asthma, the role of the epithelium seems not to be an essential step. As previously reported by Garrido-Arandia et al., Alt a 1 cannot alter the barrier integrity, being very limited its transport across the polarized bronchial cell model (less than 10%) (12). These previous studies have also shown that Alt a 1 can interact with the bronchial epithelium *via* the SLC22A17 receptor in the presence of its ligand, inducing the production of alarmins and pro-inflammatory cytokines. In contrast, this interaction does not seem to induce the Alt a 1 transport to basolateral side, but its recycling, according to the co-localization with Rab11, the most well-established mediator of endosomal recycling (36). The higher accumulation of Alt a 1 inside epithelial cells that had been previously incubated with an exocytosis inhibitor, ES-2, supported these results. This may explain why Alt a 1 is poorly detected in the basolateral side when the cell model was analyzed (6, 12). However, Alt a 1 needs to reach the basolateral side, otherwise, there would be no production of specific antibodies against this allergen and the pathway undertaken should be independent of SLC22A17.

Considering that AM represent the largest population of immune cells resident in the lung in a healthy state, Alt a 1 could be taken in by AM (F4/80+ cells) in lungs from healthy mice, and notably, co-localizing with TLR4 receptor. Previously, Hayes et al. demonstrated that IL-8 and NF-kB induction by Alt a 1 in bronchial epithelial- and embryonic kidney cell culture was mostly dependent upon TLR4 signaling, but also upon TLR2 and the adaptor proteins MyD88 and TIRAP, suggesting a key role of these receptors in Alt a 1-induce innate immune responses (6). In line with this hypothesis, we have described the Alt a 1 ability to interact with TLR4. We further confirmed in an *in vitro* monocytes culture (THP1) that the Alt a 1-induced NF-kB pathway is mediated by TLR4. The inhibition of Alt a 1-induced NF-kB signaling was complete when THP1 cells were previously incubated with TLR4 blockade antibody while the co-adaptor protein MD2 seems to be participating in this TLR4-mediated Alt a 1 recognition. Computational analyses supported this mechanism related to Alt a 1- TLR4 interaction, showing the presence of a stable complex along 100 ns MD simulations, maintained by 39 interchain hydrogen bonds. Moreover, the similarity between the final structure of the Alt a 1-TLR4 complex and the crystallographic TLR4-MD2-LPS, suggests that the presence of Alt a 1 would be able to keep the Ct-region of the extracellular domain of TLR in close contact, allowing dimerization of the intracellular domains and the activation of the TLR pathways.

Alt a 1-mediated facilitation of TLR4 signaling in the presence of very low (or no) LPS exposure may promote the T2 response. Similarly, Alt a 1 probably promotes the development or exacerbation of asthma, possibly due to its ability to interact with TLR4 on alveolar macrophages in the airways. In this case, the ability of Alt a 1 to reconstitute TLR4 signaling and activation, in the absence of ligand, may be of particular importance. Indeed, the structural similarity between MD2 and Alt a 1 would only explain why Alt-1 can interact with TLR4, but not why it can activate the TLR4 activation cascade without ligand. This fact highlights the ability of a protein to activate the immune system towards an asthma phenotype without the need of adjuvant activity or associated ligands as has been described for other proteins.

**Figure 8:**
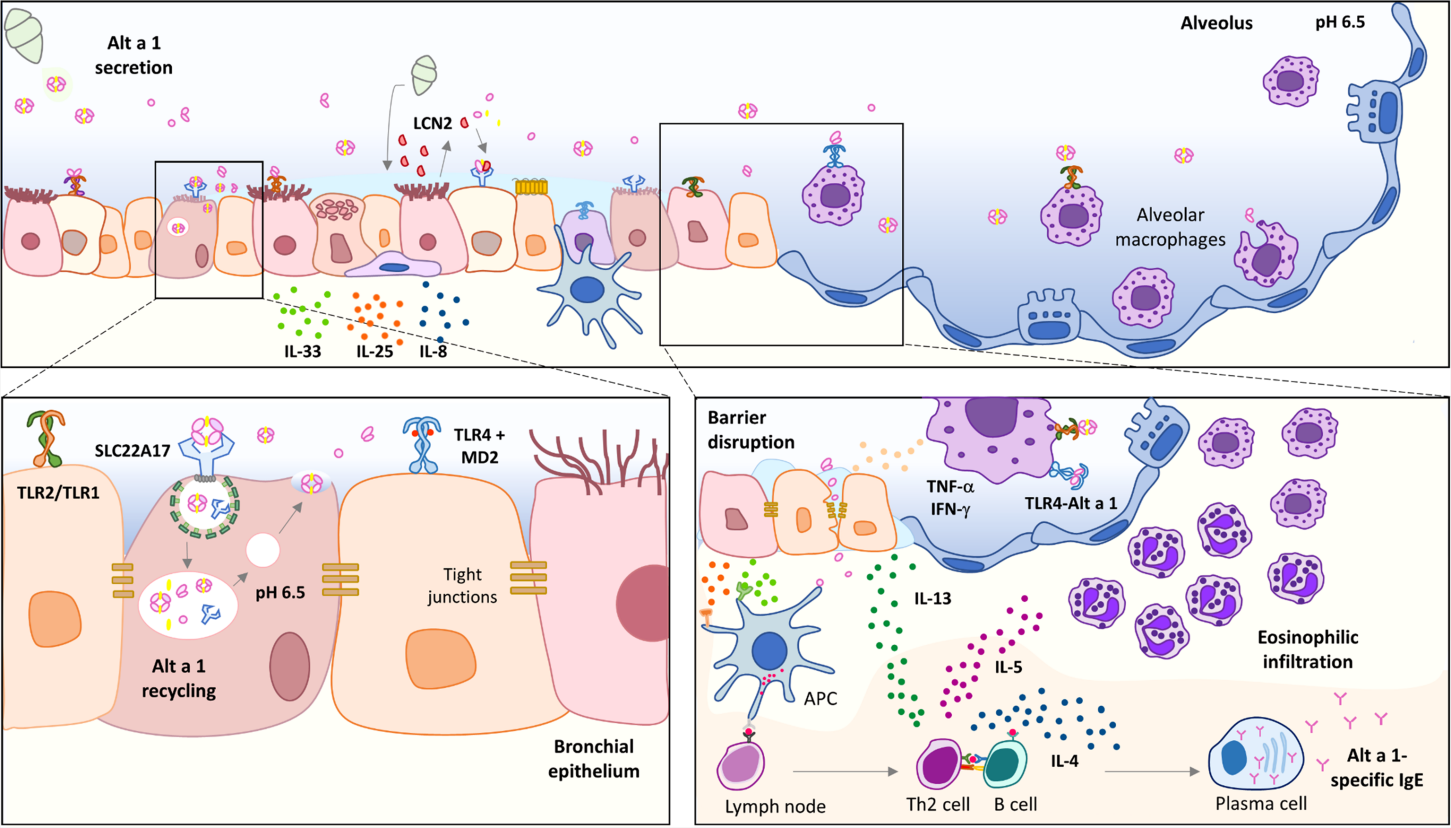
Proposed model for Alt a 1 sensitization.

In summary, this study has highlighted two events: first, the description of the ability of Alt a 1 to induce allergic airway inflammation in a mouse model regardless of the presence of a ligand; and, second, the potential role of lung macrophages mediating the Alt a 1-immune responses (**Figure 8**). Although the interaction of Alt a 1 with TLR4 is unlikely to be of biological importance to the mold, it is tempting to speculate that Alt a 1 plays an essential role in establishing the ideal conditions for the fungus development. The pathophysiological interactions of Alt a 1 with the immune system may be more than a unique oddity.

## Data Availability Statement

The original contributions presented in the study are included in the article/**Supplementary Materials**, further inquiries can be directed to the corresponding author/s.

## Ethics Statement

The animal study was reviewed and approved by Institutional Animal Care and Use Committee from Community of Madrid.

## Author Contributions

ADP, JTA, MGA and GHR designed the study. GHR, DPC, ZGK, JLRG, SFB, LPG, and JTA performed experimental assays. Data was analyzed by GHR, DPC, ZGK, JTA, VE, and ADP. Manuscript was written and revised by GHR, JTA and ADP. All authors reviewed and approved the manuscript.

## Funding

This research was funded by the Spanish Ministry of Science and Innovation through the project LISENTRA, granted by the Spanish Research State Agency (PID2020-113629RB00/AEI/10.13039/501100011033). GH-R was granted by funding from the European Commission through the project AllerScreening, granted within the R&D framework programme Horizon2020 (H2020-NMBP-X- KET-2017-768641). DP-C was granted by Universidad Politécnica de Madrid and Banco Santander for a predoctoral Programa Propio grant. JT-A and ZG-K were granted by funding from the Community of Madrid included in the project FOODAL (FOODAL-CM; S2018/BAA-4574) co-funded by ESF and ERDF R&D projects call Tecnologías 2018. This work was also supported by Instituto de Salud Carlos III (ISCIII) co-funded by FEDER Thematic Networks and Cooperative Research Centers: ARADYAL (RD16/0006/0003; RD16/0006/0013).

## Conflict of Interest

The authors declare that the research was conducted in the absence of any commercial or financial relationships that could be construed as a potential conflict of interest.

## Publisher’s Note

All claims expressed in this article are solely those of the authors and do not necessarily represent those of their affiliated organizations, or those of the publisher, the editors and the reviewers. Any product that may be evaluated in this article, or claim that may be made by its manufacturer, is not guaranteed or endorsed by the publisher.
