# Supplementary material for: Alt a 1 Promotes Allergic Asthma In Vivo Through TLR4-Alveolar Macrophages
Source: Front Immunol. 2022 Jun 30;13:877383. doi: 10.3389/fimmu.2022.877383 (PMC9280186; doi:10.3389/fimmu.2022.877383)
Supplement: Supplementary file 1 [file Image_1.pdf]

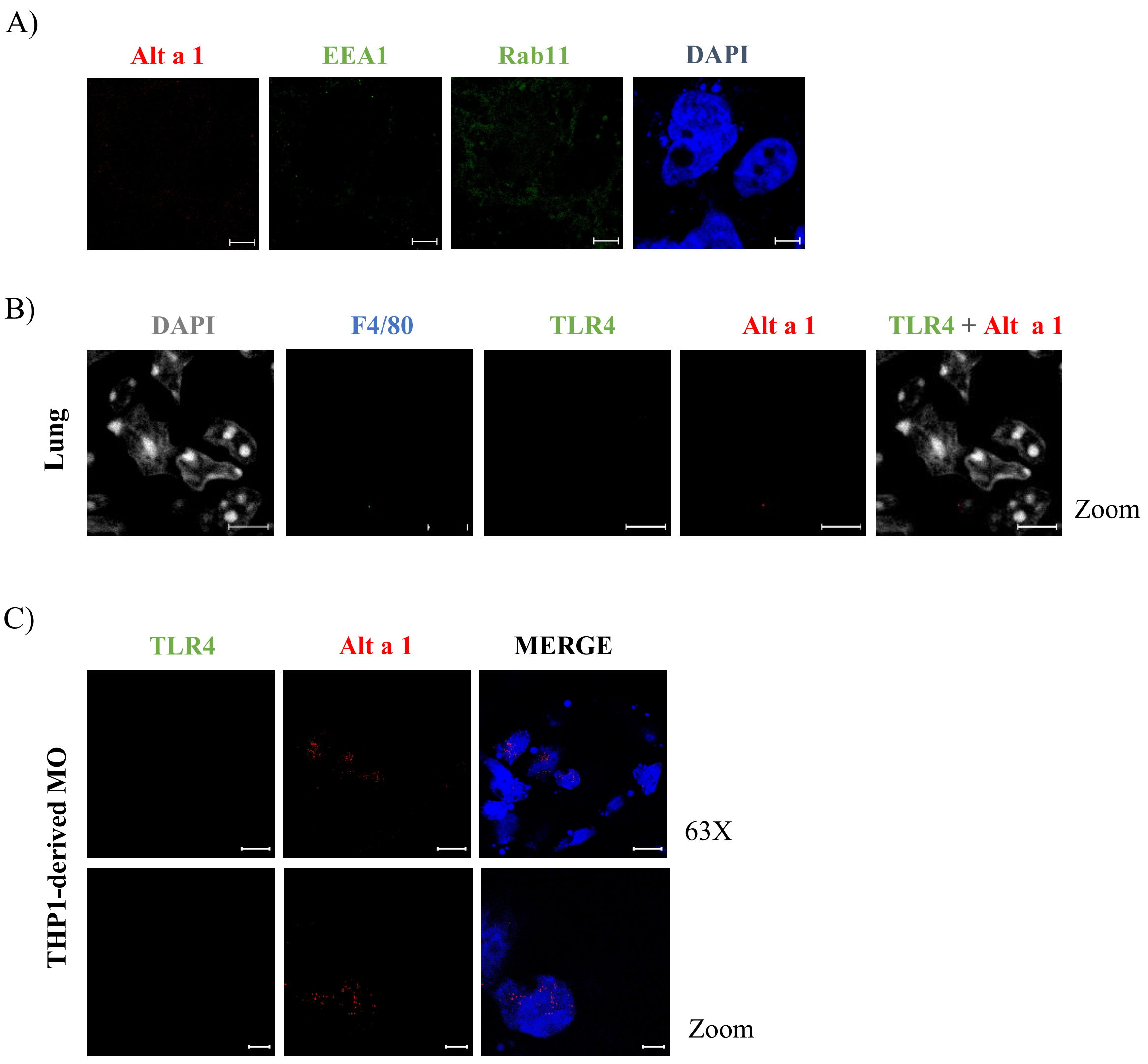

Supplementary Fig. 1. A) Immunolocalization of Alt a 1 with endosome markers in Calu-3 cells. Isotype controls are shown in the image for Alt a 1, EEA1 and Rab11 detections. Scale bar = 5 um. **Immunolocalization of Alt a 1 with TLR4 in macrophages.** Isotype controls are shown in the images for each immunofluorescence assay: (B) co-localization of Alt a 1 and TLR4 in F4/80^{+} lung cells (bar = 5 µm); and (C) co-localization of Alt a 1 and TLR4 in THP1-derived macrophages (scale bar for .63X magnification is 20 µm and 5 µm for zoom captures)
