# Supplementary material for: Alt a 1 Promotes Allergic Asthma In Vivo Through TLR4-Alveolar Macrophages
Source: Front Immunol. 2022 Jun 30;13:877383. doi: 10.3389/fimmu.2022.877383 (PMC9280186; doi:10.3389/fimmu.2022.877383)
Supplement: Supplementary file 2 [file Table_1.pdf]

Supplementary Table 1. List of primary antibodies

.

Target	Reference/clone	Conjugated	Use dilution
Alt a 1	Pab (Roxall)	-	1/1000
IgG1	A90-105A (Bethyl laboratories)	-	1/10000
IgG2a	A90-107A (Bethyl laboratories)	-	1/10000
IgE	PA-184764 (Fisher scientific)	HRP	1/2000
Siglec-F	Clone E50-2440 (BD Biosciences)	Brilliant Violet 421 nm	1/300
F4/80	Clone BM8 (Invitrogen)	FITC / Alexa Fluor 488	1/50
CD3	Clone 17A2 (eBioscience)	Alexa Fluor 555	1/100
CD45	Clone 30-F11 (eBioscience)	Alexa Fluor 555	1/100
Ly6G	1AB-Ly6g (Invitrogen)	PE-eFluor 610	1/100
ORDML3	ABN417 (Merck Millipore)	-	1/1000
Caspase-1	22915-1-AP (Proteintech)	-	1/2000
β-Tubulin	10068-1-AP (Proteintech)	-	WB: 1/2000
Rab 11a	AB0034-200 (Sicgen)	-	1/400
EEA1	Clone EEA1-N19 (Sigma-Aldrich)	-	1/400
TLR4	PA5-23125 (Invitrogen)	-	1/100
TLR4*	PAb-hTLR4(Invivogen)	-	1/100

*Antibody indicated for TLR-mediated signaling inhibition.
